# Clinical and Inflammatory Correlates of Fractional Exhaled Nitric Oxide in Turkish Children and Adolescents with Asthma: Exploratory Analysis of the FCER2 rs2228137 Polymorphism

**DOI:** 10.3390/children13101291

**Published:** 2026-09-23

**Authors:** Azer Kilic Baskan, Berrak Oztosun, Deniz Ozceker, Aysel Kalayci, Mehmet Seven, Haluk Cokugras, Ayse Ayzıt Kilinc Sakallı

**Affiliations:** 1 Department of Pediatric Pulmonology, Cerrahpasa Faculty of Medicine, Istanbul University—Cerrahpasa, 34098 Istanbul, Türkiye; azer.kilicbaskan@iuc.edu.tr (A.K.B.);; 2 Department of Pediatric Allergy and Immunology, University of Health Sciences, Cemil Tascioglu City Hospital, 34384 Istanbul, Türkiye; 3 Department of Medical Genetics, Cerrahpasa Faculty of Medicine, Istanbul University—Cerrahpasa, 34098 Istanbul, Türkiye

**Keywords:** adolescent, asthma, child, FCER2, fractional exhaled nitric oxide, polymorphism

## Abstract

**Highlights:**

**What are the main findings?**

- In inhaled corticosteroid/long-acting β-agonist-treated children and adolescents with asthma, blood eosinophil percentage and symptom-based control (ACQ score) were independently associated with FeNO in a multivariable model adjusted for prespecified baseline covariates (age, sex, BMI z-score), whereas total IgE and aeroallergen sensitization were not; descriptive gradients across ATS FeNO categories were observed for several type 2 markers.
- The exploratory FCER2 rs2228137 (R62W) polymorphism, examined as a covariable in the adjusted models, was not associated with FeNO or related clinical and inflammatory phenotypes under codominant, additive, dominant or recessive coding (hypothesis-generating; TT genotype, *n* = 5).

**What are the implications of the main findings?**

- FeNO remains a useful noninvasive marker of type 2 airway inflammation even in children and adolescents already receiving controller therapy, and is best interpreted together with blood eosinophils, total IgE, sensitization and symptom scores rather than in isolation.
- Routine rs2228137 genotyping is not supported for asthma phenotyping in this population; larger longitudinal, multicenter studies are needed to clarify any context-specific role.

**Abstract:**

**Background/Objectives:** Fractional exhaled nitric oxide (FeNO) is a noninvasive biomarker of eosinophilic airway inflammation in asthma, but data on its clinical correlates in Turkish children and adolescents are limited. This study aimed to determine which clinical and inflammatory characteristics were independently associated with FeNO and, as a secondary exploratory objective, to assess whether the FCER2 rs2228137 (R62W) polymorphism is associated with FeNO and related clinical features. **Methods:** In this cross-sectional study of 100 children and adolescents aged 6–18 years with asthma receiving inhaled corticosteroid/long-acting beta-agonist therapy at a tertiary center in Istanbul, Türkiye, FeNO, spirometry, skin prick testing, peripheral blood eosinophil percentage, total IgE, and Asthma Control Questionnaire (ACQ) score were measured. Genotyping of rs2228137 was performed by SNaPshot multiplex assay and confirmed by Sanger sequencing. The primary inferential analysis was a multivariable linear regression of ln-transformed FeNO with age, sex and BMI z-score as prespecified baseline covariates; analyses by American Thoracic Society (ATS) FeNO categories were descriptive, and genotype was examined as a covariable in adjusted regression models. **Results:** In the primary multivariable analysis (*n* = 98; R^2^ = 0.42), blood eosinophil percentage (β = 0.082, 95% CI 0.039–0.124; *p* < 0.001) and ACQ score (β = 0.264, 95% CI 0.135–0.394; *p* < 0.001) were independently associated with ln(FeNO), as was age (β = 0.074, 95% CI 0.013–0.134; *p* = 0.02), whereas total IgE and aeroallergen sensitization were not independently associated with FeNO. Descriptive analyses across clinically defined ATS FeNO categories showed gradients consistent with a greater type 2 inflammatory burden and poorer symptom control at higher FeNO levels. In exploratory analyses with the rs2228137 genotype entered as a covariable in the adjusted models, no genotype–phenotype association was statistically significant across the examined models (FeNO, FEV1, eosinophil percentage, total IgE, skin prick test positivity, uncontrolled asthma); this prespecified secondary analysis was statistically underpowered, particularly for the TT genotype (*n* = 5). **Conclusions:** FeNO is a useful adjunctive biomarker of type 2 airway inflammation and symptom control in Turkish children and adolescents with asthma, whereas the exploratory FCER2 rs2228137 analysis did not reveal genotype–phenotype associations; given the small subgroup sizes, these findings should be regarded as hypothesis-generating.

## 1. Introduction

Asthma is one of the most common chronic respiratory diseases in children and remains a major cause of school absences, emergency department visits, and hospital stays. Its clinical course varies widely, with significant differences in severity, exacerbation frequency, and treatment response, likely due to variations in genetic background, environmental exposures, and underlying immunologic mechanisms [1,2]. In a significant subset of children and adolescents, asthma is characterized by type 2-high eosinophilic airway inflammation, reflected by elevated peripheral blood eosinophils, increased total immunoglobulin E (IgE), aeroallergen sensitization, and increased fractional exhaled nitric oxide (FeNO) [3].

FeNO is a practical, noninvasive biomarker of eosinophilic airway inflammation and likely corticosteroid responsiveness [4]. Nitric oxide is produced by inducible nitric oxide synthase in the airway epithelium, and its expression is upregulated by type 2 cytokines, particularly IL-4 and IL-13 [5]. Accordingly, FeNO correlates with eosinophilic inflammation, decreases with effective inhaled corticosteroid (ICS) treatment, and may increase with poor adherence or persistent airway inflammation [6]. In pediatric populations, FeNO consistently correlates with peripheral blood eosinophil percentages, total IgE levels, and aeroallergen sensitization, supporting its role as a meaningful clinical surrogate for the type 2 inflammatory profile [7]. Evidence also connects elevated FeNO to impaired lung function and poor asthma control, indicating that FeNO can help monitor disease burden beyond spirometry [8]. However, population-specific data from Turkish children and adolescents on ICS-based therapy remain limited. Turkish pediatric studies on FeNO have largely examined it in specific contexts, such as its relationship with serum periostin or in the setting of obesity and exercise, rather than its association with asthma control and allergic-inflammatory markers in ICS-treated children and adolescents [9,10]. Beyond inflammatory biomarkers, genetic factors influencing asthma-related phenotypes have also been studied. The FCER2 gene encodes CD23, a low-affinity IgE receptor on B lymphocytes that is involved in the feedback regulation of IgE production [11]. The rs2228137 (R62W) single-nucleotide polymorphism has been investigated as a potential modifier of asthma phenotype, but reported genotype–phenotype links have been inconsistent. Differences in ethnicity, treatment exposure, and sample size may partly explain these conflicting results. Data on the FCER2 rs2228137 polymorphism in Turkish children and adolescents with asthma, especially in connection with FeNO-based inflammation profiles, are limited.

Therefore, the primary objective of this study was to determine whether peripheral blood eosinophil percentage, total IgE, aeroallergen sensitization, and asthma control were independently associated with FeNO in Turkish children and adolescents with asthma receiving ICS-based therapy. Lung function was additionally characterized descriptively in relation to FeNO, and FeNO was described according to the clinically established ATS categories. As an exploratory objective, we assessed whether the FCER2 rs2228137 polymorphism was associated with FeNO and related clinical and inflammatory features.

## 2. Materials and Methods

### 2.1. Study Design and Participants

This cross-sectional study was conducted at the Pediatric Pulmonology Department. Children and adolescents aged 6–18 years with a diagnosis of asthma established according to GINA criteria were consecutively enrolled [12]. Recruitment took place between April 2021 and February 2025. All participants were receiving combination inhaled corticosteroid (ICS) and long-acting beta-agonist (LABA) therapy at enrollment; the ICS daily-dose category (low/medium/high) and treatment duration were recorded from the clinical record. Exclusion criteria included chronic respiratory diseases other than asthma, cardiovascular disease, active systemic infection, autoimmune or autoinflammatory disease, primary or secondary immunodeficiency, and malignancy. Patients younger than 6 years or older than 18 years, and those whose parents or legal guardians did not provide written informed consent, were also excluded. The study was approved by the Ethics Committee of Istanbul University—Cerrahpasa, Cerrahpasa Faculty of Medicine (approval number: 78916; approval date: 16 April 2021). Written informed consent was obtained from all participants and their parents or legal guardians in accordance with the Declaration of Helsinki.

### 2.2. Clinical Data Collection

Demographic data, medical and family history, and clinical examination findings were recorded for all patients. We systematically collected the following parameters: FeNO levels, spirometric measurements, peripheral blood eosinophil percentage, serum total IgE, allergen skin prick test (SPT) results, and Asthma Control Questionnaire (ACQ) scores. Asthma control was assessed using the seven-item Asthma Control Questionnaire (ACQ), validated for children aged 6 years and older. Based on ACQ scores, patients were classified as well-controlled (ACQ ≤ 0.75), partially controlled (ACQ > 0.75–≤ 1.50), or uncontrolled (ACQ > 1.50).

The ACQ [13] was interviewer-administered using an official Turkish version provided by the copyright holder (linguistically validated). Because pre-bronchodilator FEV1 is one of the seven ACQ items, FEV1 was not additionally described across the ACQ-defined control groups so as to avoid circularity (see Section 3.4).

### 2.3. FeNO Measurement

FeNO concentration was measured using the NObreath^®^ FeNO monitor (Bedfont Scientific, Maidstone, UK) at a constant expiratory flow rate of 50 mL/s for 6 s. The mean value of two technically acceptable measurements was used for analysis. FeNO levels were categorized according to American Thoracic Society (ATS) pediatric thresholds as normal (<20 ppb), intermediate (20–35 ppb), or high (>35 ppb) [14].

Because the cohort predominantly comprised children and younger adolescents (median age 12 years; 42% younger than 12 years), the ATS paediatric decision points were used for the descriptive categorization of FeNO. FeNO was measured before spirometry in all participants.

### 2.4. Pulmonary Function Testing

Spirometric measurements were performed by a single trained technician using the MasterScreen PFT system (Jaeger, Würzburg, Germany) with Desktop Sentry-Suite v2.19 software. All procedures followed the ATS/ERS Standardization of Spirometry guidelines. FEV1 and FVC were expressed as percentages of predicted values.

### 2.5. Allergic Sensitization Assessment

Allergen sensitization was evaluated by SPT using a standardized panel of 11 aeroallergens: Alternaria alternata, cat dander, dog dander, Dermatophagoides farinae, Dermatophagoides pteronyssinus, tree mixture, grass mixture, weed mixture, olive, Parietaria, and rye. Histamine (1 mg/mL) and 0.9% saline served as positive and negative controls, respectively. A wheal diameter at least 3 mm greater than that of the negative control was considered positive.

### 2.6. Laboratory Analyses

The percentage of peripheral blood eosinophils was determined by automated complete blood count analysis. Serum total IgE concentration was measured by chemiluminescence.

### 2.7. FCER2 Genotyping

As a secondary exploratory analysis, the FCER2 rs2228137 (R62W) polymorphism was analyzed at the GETAM Laboratory, Cerrahpasa Faculty of Medicine. We isolated DNA from 2 mL of peripheral venous blood collected during routine sampling. After DNA amplification using Multiplex PCR Master Mix, genotyping was performed using the SNaPshot Multiplex Kit (Applied Biosystems, Thermo Fisher Scientific, Foster City, CA, USA). Sanger sequencing confirmed genotype calls.

Genotyping quality control was satisfactory, with a 100% call rate for rs2228137, 100% duplicate concordance, no amplification in the no-template controls, and Sanger confirmation of all 100 samples. The variant is annotated according to HGVS nomenclature as NM_002002.5:c.184C>T (p.Arg62Trp; rs2228137), reported against genome build GRCh38. The variant allele is denoted as T on the transcript strand; studies reporting it as G refer to the opposite strand, and our genotype calls are aligned accordingly. Genotype–phenotype associations were examined by entering the genotype as a covariable in adjusted regression models (Section 2.8) under codominant (primary), additive (allele-dose), dominant and recessive coding.

### 2.8. Statistical Analysis

Statistical analyses were performed using IBM SPSS Statistics for Windows, version 26.0 (IBM Corp., Armonk, NY, USA); the genotype-covariable models were fitted in Python 3.11 (SciPy 1.17). Continuous variables are summarized as mean ± standard deviation or median with interquartile range (IQR), as appropriate, and categorical variables as number (%). The primary inferential analysis was based on multivariable linear regression with natural-log-transformed FeNO as the dependent variable. Blood eosinophil percentage, ln-transformed total IgE, skin prick test positivity and ACQ score were entered as the clinical/inflammatory variables of interest, with age, sex and BMI z-score included as prespecified baseline covariates selected. Regression coefficients (β) with 95% confidence intervals (CIs) were reported, and collinearity was assessed using variance inflation factors (VIFs). Because FEV1 is a component of the ACQ, it was not entered in the same model. Analyses based on the clinically defined ATS FeNO categories and on the ACQ-defined control categories were retained as descriptive analyses to facilitate clinical interpretation of the established thresholds; no separate between-group significance tests are reported, and these analyses were not considered the basis for inference. Likewise, unadjusted correlations were not treated as independent confirmatory tests, and the previously reported rank correlations have been omitted; Figure 1 shows the corresponding relationships for descriptive purposes only.

The FCER2 rs2228137 analyses were prespecified as exploratory because of the small number of TT homozygotes. Genotype was entered as a covariable in regression models adjusted for age, sex and BMI z-score, using linear regression for ln(FeNO), FEV1, eosinophil percentage and ln total IgE, and logistic regression for skin prick test positivity and uncontrolled asthma (ACQ > 1.5); genotype was coded codominantly (TC and TT versus CC, with a 2-df Wald test for the overall genotype effect; primary) and, additionally, as an additive allele-dose term (0, 1 or 2 T alleles) and under dominant (T-carrier versus CC) and recessive (TT versus C-carrier) coding. Hardy–Weinberg equilibrium for rs2228137 was tested by a χ^2^ goodness-of-fit test. A two-sided *p*-value of <0.05 was considered statistically significant; given the exploratory nature of the genotype analyses, no correction for multiple testing was applied and emphasis was placed on effect estimates and confidence intervals rather than on isolated *p*-values. No a priori sample-size calculation was performed, as all eligible consecutive patients presenting during the recruitment window were enrolled; post hoc, 100 participants provided at least 80% power (α = 0.05, two-sided) to detect a standardized regression coefficient of |β| ≥ 0.28. As a sensitivity analysis retained from the previous revision, the associations of the type 2 markers and the ACQ score with FeNO category were additionally examined with proportional-odds (ordinal logistic) regression, categorizing FeNO at the pediatric (20 and 35 ppb) and at the adult (25 and 50 ppb) ATS decision points, unadjusted and adjusted for age, sex and BMI z-score (Supplementary Table S1).

## 3. Results

### 3.1. Baseline Characteristics of the Study Population

One hundred children and adolescents with asthma aged 6–18 years (mean age, 12.2 ± 3.3 years; median 12 years, with 42% younger than 12 years) were enrolled. The cohort included 60 boys (60%) and 40 girls (40%), with a mean BMI of 20.13 ± 4.04 kg/m^2^. A history of atopy was present in 55% of participants, a family history of asthma in 40%, and prenatal cigarette smoke exposure in 48%. The mean peripheral blood eosinophil percentage was 6.49 ± 4.81%, and the median total IgE level was 289 U/L (IQR: 67–851 U/L). The median FeNO level was 35.0 ppb (IQR: 10.0–88.0 ppb). FEV1 and FVC were 100.01 ± 15.83% and 98.21 ± 16.28%, respectively. SPT positivity for aeroallergens was detected in 74% of patients. Baseline characteristics are summarized in Table 1.

The inhaled corticosteroid daily-dose category was low in 3 patients (3%), medium in 39 (39%) and high in 58 (58%), and the median treatment duration was 24 months (IQR 2–36).

### 3.2. Primary Multivariable Analysis of FeNO

In the primary multivariable linear regression of ln(FeNO) (Table 2; *n* = 98; R^2^ = 0.42), blood eosinophil percentage (β = 0.082, 95% CI 0.039 to 0.124; *p* < 0.001) and ACQ score (β = 0.264, 95% CI 0.135 to 0.394; *p* < 0.001) were independently associated with FeNO after adjustment for the other variables in the model, as was age (β = 0.074, 95% CI 0.013 to 0.134 per year; *p* = 0.02). Total IgE (*p* = 0.39) and skin prick test positivity (*p* = 0.82) were not independently associated with FeNO, and sex and BMI z-score were not associated. Variance inflation factors were all below 1.5. The adjusted estimates are displayed as a forest plot in Figure 2; the prespecified conceptual framework used to select the covariates is depicted in Figure 3.

**Figure 2:**
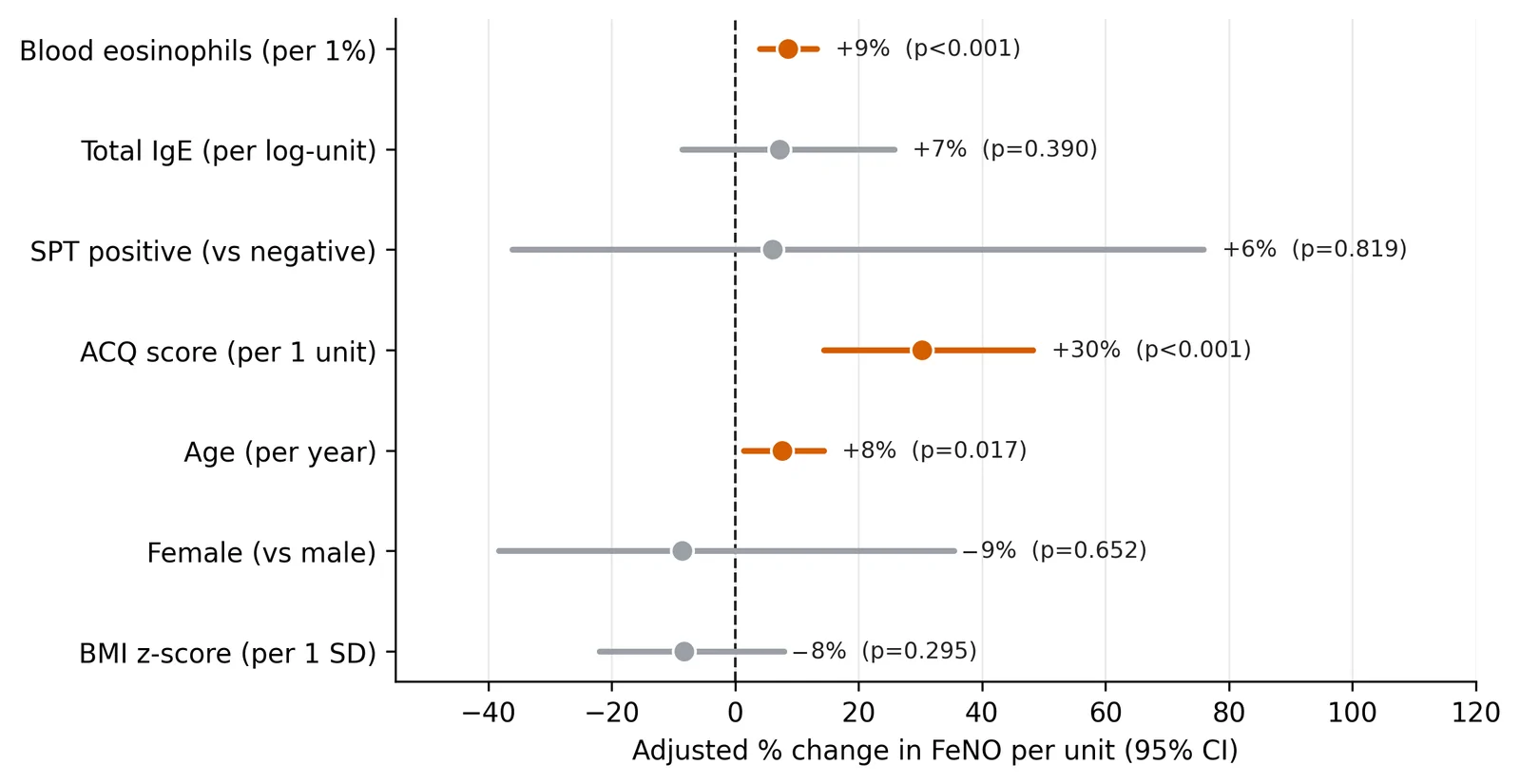
Forest plot of the primary multivariable linear regression of ln(FeNO) (Table 2; *n* = 98; R^2^ = 0.42): adjusted percentage change in FeNO per unit of each predictor with 95% confidence intervals. Coloured markers indicate *p* < 0.05.

**Figure 3:**
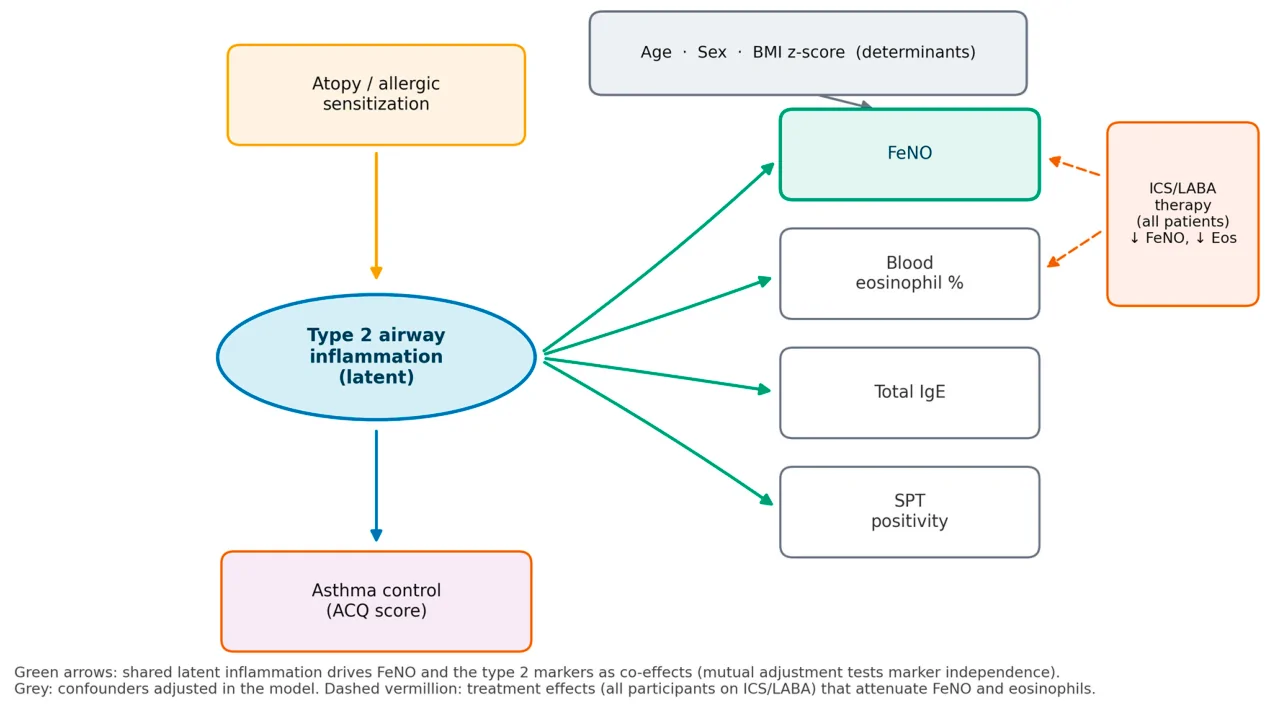
Prespecified conceptual framework, drawn as a directed acyclic graph, used to inform covariate selection for the FeNO analysis. FeNO, blood eosinophils, total IgE and skin-prick positivity are represented as correlated markers of a shared latent type 2 airway-inflammation process; age, sex and BMI are baseline determinants of FeNO and were prespecified as baseline covariates, and ICS/LABA therapy (received by all participants) attenuates FeNO and eosinophils. The framework was used only to inform covariate selection and does not imply causal inference from this cross-sectional dataset.

### 3.3. Descriptive Analysis According to ATS FeNO Categories

For clinical interpretation, patients were stratified according to the ATS pediatric decision points into normal (<20 ppb; *n* = 40), intermediate (20–35 ppb; *n* = 10) and high (>35 ppb; *n* = 50) FeNO categories (Table 3). Descriptively, the high-FeNO group had the highest mean peripheral blood eosinophil percentage (7.8 ± 5.1% versus 5.0 ± 4.3% in the normal-FeNO group), the highest median total IgE (443 versus 157.5 U/L), the highest proportion of SPT positivity (84.0% versus 62.5%) and the highest median ACQ score (2.10 versus 0.65), whereas FEV1 was similar across categories. The rs2228137 TT genotype was present in 1, 2 and 2 patients in the three categories, respectively. Descriptive gradients were thus observed for several type 2 inflammatory markers; however, only blood eosinophil percentage and ACQ score remained independently associated with FeNO in the primary multivariable model (Table 2). In accordance with the analysis plan, no separate between-group tests were performed. The relationships of FeNO with blood eosinophils, total IgE and ACQ score are illustrated in Figure 1. In the threshold sensitivity analysis (Supplementary Table S1), blood eosinophil percentage, total IgE and ACQ score were associated with a higher FeNO category at both the pediatric and the adult decision points after adjustment for age, sex and BMI z-score, whereas the association of SPT positivity was threshold-dependent and attenuated after adjustment (adjusted OR per category 2.27 (0.89–5.79) at the pediatric and 1.63 (0.64–4.16) at the adult decision points).

### 3.4. Descriptive Analysis According to Asthma Control Categories

Asthma control status was classified from the ACQ score as well-controlled in 32 patients (32%), partially controlled in 30 (30%), and uncontrolled in 38 (38%) (Table 4). Descriptively, median FeNO was lowest in the well-controlled group (16 ppb) and higher in the partially controlled (35 ppb) and uncontrolled (44 ppb) groups, and SPT positivity increased across the control categories (59.4%, 70.0% and 89.5%), whereas peripheral blood eosinophil percentage and total IgE were similar. The rs2228137 TT genotype was present in 1, 1 and 3 patients, respectively. These descriptive patterns are consistent with the independent association between the ACQ score and FeNO in the primary model (Table 2); no separate between-group tests were performed.

### 3.5. Exploratory Analysis of the FCER2 rs2228137 Genotype

Among the 100 patients, 60 (60%) had the homozygous CC genotype, 35 (35%) had the heterozygous TC genotype, and 5 (5%) had the homozygous TT genotype of rs2228137; their clinical and laboratory characteristics are summarized in Table 5. With genotype entered as a covariable in the regression models adjusted for age, sex and BMI z-score, no association was observed under the primary codominant coding with FeNO (overall *p* = 0.61), FEV1 (*p* = 0.61), peripheral blood eosinophil percentage (*p* = 0.62), total IgE (*p* = 0.71), SPT positivity (*p* = 0.59) or uncontrolled asthma (*p* = 0.59), and results were similar under additive, dominant and recessive coding (all *p* ≥ 0.30). Although median total IgE was numerically highest in the five TT homozygotes (997 U/L), the adjusted TT-versus-CC difference in ln(total IgE) was small and imprecise (β = −0.08, 95% CI −1.42 to 1.26; *p* = 0.91). These exploratory results are summarized in Table 5.

The rs2228137 genotype distribution was in Hardy–Weinberg equilibrium (χ^2^ = 0.0013, df = 1, *p* = 0.97; exact test *p* = 1.00), with an observed T-allele frequency of 0.225 (95% CI 0.173–0.288). This is appreciably higher than the frequency reported for East Asian populations using the same variant notation and genotyping platform (0.07 in Chinese Han healthy controls and 0.10 in Chinese asthmatic patients [15]), consistent with a population-specific distribution of rs2228137. For FeNO specifically, the adjusted genotype effects were null under codominant coding (TC versus CC: β = −0.25, 95% CI −0.75 to 0.25; TT versus CC: β = −0.14, 95% CI −1.21 to 0.94; overall *p* = 0.61), additive coding (β = −0.17, 95% CI −0.56 to 0.23 per T allele; *p* = 0.40), dominant coding (*p* = 0.33) and recessive coding (*p* = 0.94).

## 4. Discussion

The principal finding of this cross-sectional study of Turkish children and adolescents with asthma was that peripheral blood eosinophil percentage and symptom-based asthma control (ACQ score) remained independently associated with FeNO in the multivariable model adjusted for prespecified baseline covariates, whereas total IgE and aeroallergen sensitization did not. The descriptive analyses across clinically defined FeNO categories showed broadly consistent patterns—higher eosinophil percentages, total IgE, sensitization rates and ACQ scores at higher FeNO levels—but should be regarded as supportive rather than as independent inferential findings. Together, these results support the use of FeNO as an additional marker of type 2 airway inflammation in this population. In contrast, the secondary exploratory analysis of the FCER2 rs2228137 polymorphism did not reveal genotype–phenotype associations; given the small number of TT homozygotes (*n* = 5) and the limited statistical power, these exploratory findings should be regarded as hypothesis-generating and do not exclude a true association.

FeNO is widely recognized as a noninvasive marker of eosinophilic airway inflammation, and our findings are consistent with this idea. The independent association of FeNO with blood eosinophil percentage in the adjusted model (approximately 9% higher FeNO per 1% increase in eosinophils), together with the descriptive gradient of total IgE and aeroallergen sensitization across FeNO categories, indicates a consistent type 2 inflammatory response profile. Importantly, these associations remained detectable despite background ICS/LABA therapy in all participants, indicating that FeNO may still retain clinical relevance in children and adolescents already receiving controller treatment. This is particularly important in routine practice, where biomarker interpretation usually occurs in treated rather than treatment-naïve patients.

In the multivariable model, blood eosinophil percentage and symptom control (ACQ) remained independently associated with FeNO, whereas total IgE and aeroallergen sensitisation did not, indicating that these type 2 markers convey overlapping rather than fully independent information. Age was also independently associated with FeNO, consistent with the known increase in FeNO with age and height in children, which supports its inclusion as a prespecified baseline covariate. Consistently, in the threshold sensitivity analysis the association of aeroallergen sensitization with FeNO category was threshold-dependent and attenuated after adjustment, whereas blood eosinophils, total IgE and ACQ score were robust to the choice of ATS decision points (Supplementary Table S1).

The associations between FeNO and peripheral blood eosinophils, total IgE, and aeroallergen sensitization observed in our cohort are consistent with prior reports in pediatric populations. Our findings also extend the limited Turkish pediatric literature on FeNO, which has previously focused on periostin-related analyses and obesity/exercise settings rather than routine clinical-inflammatory correlates in ICS-treated children and adolescents [9,10]. Cardinale et al. demonstrated significant correlations between FeNO, serum IgE, and allergic sensitization in children with asthma and allergic rhinitis, supporting the shared type 2 pathway underlying these markers [16]. Similarly, Zhu et al. reported that atopic sensitization and blood eosinophils were among the strongest determinants of elevated FeNO in children with asthma and allergic rhinitis [17]. Strunk et al. further demonstrated that FeNO was associated with both clinical and inflammatory markers of asthma persistence in children, including blood eosinophilia and IgE [18]. In line with these findings, our data support the ATS recommendation that FeNO be interpreted in conjunction with clinical and inflammatory parameters rather than as a standalone marker [14]. Regarding asthma control, the independent association between the ACQ score and FeNO in the adjusted model, together with the descriptively lower FeNO in well-controlled patients, is consistent with evidence that elevated FeNO reflects ongoing airway inflammation associated with suboptimal symptom control [17]. The absence of a FeNO–FEV1 association in our cohort may reflect the well-described dissociation between eosinophilic airway inflammation and fixed airflow limitation, particularly in ICS-treated patients in whom spirometric values may remain preserved despite ongoing mucosal inflammation [14,19].

Our results also suggest that airway inflammation, lung function, and symptom-based control reflect related but not identical dimensions of disease. FeNO was independently associated with the ACQ score, supporting a relationship between FeNO and clinical control, whereas FEV1 was similar across FeNO categories. This pattern indicates that FeNO, spirometry, and symptom-based questionnaires capture overlapping but distinct aspects of asthma activity. Conversely, symptoms and questionnaire scores may be influenced by factors other than current eosinophilic airway inflammation, such as adherence, inhaler technique, upper-airway comorbidity, and behavioral factors. Overall, these findings support using FeNO as a complementary rather than interchangeable assessment tool.

From a clinical perspective, these observations are meaningful. Childhood and adolescence are periods in which treatment adherence may be inconsistent, symptom perception may be variable, and lung function alone may not fully reflect ongoing airway inflammation. In this setting, FeNO offers a rapid, noninvasive marker that may help identify a type 2-high inflammatory profile when interpreted alongside peripheral blood eosinophils, total IgE, aeroallergen sensitization, and symptom-based assessment. Our data do not suggest that FeNO should replace standard clinical evaluation; rather, they support a multimodal approach in which FeNO contributes biologic information beyond symptoms and spirometry alone. This may be especially useful in children and adolescents with discordant clinical features, such as relatively preserved spirometry despite ongoing inflammatory activity or disproportionate symptoms in the setting of modest objective findings.

The secondary exploratory analysis of FCER2 rs2228137 did not yield clinically informative associations in this cohort. With genotype entered as a covariable in the adjusted regression models, no association was observed with FeNO, FEV1, peripheral blood eosinophil percentage, total IgE, SPT positivity, or uncontrolled asthma under codominant, additive, dominant or recessive coding. Although median total IgE was numerically highest in TT homozygotes, only five patients carried this genotype, the adjusted estimate was imprecise, and this isolated observation should not be overinterpreted. The absence of significant findings may reflect limited statistical power, treatment-related suppression of inflammatory markers by ICS therapy, or true population-specific differences. The rs2228137 (R62W) variant has been shown to confer resistance of CD23 to enzymatic cleavage in cell culture models, potentially leading to sustained surface expression and altered IgE feedback regulation [20]. Chan et al. reported that this SNP was associated with increased IL-4 receptor expression in human B cells, suggesting a plausible mechanism for genotype-related differences in IgE biology [21]. Despite this molecular evidence, clinical associations have been inconsistent. Zheng et al. reported mixed associations across populations of different ethnic backgrounds [15], and Potaczek et al. noted that the functional impact of FCER2 variants may depend on haplotype context and population-specific linkage disequilibrium patterns [22]. Taken together, these observations suggest that the clinical relevance of rs2228137 is context-dependent, and that our null findings in a Turkish cohort of children and adolescents may reflect true population-level heterogeneity in addition to limited statistical power. It should also be emphasized that our study included only children and adolescents with asthma and no healthy controls; therefore, it was not designed to evaluate genotype-related disease susceptibility. In the context of the present study, rs2228137 should be regarded as an exploratory genetic variable rather than a clinically useful biomarker.

Another important point is that our findings position FeNO more clearly within a clinical–immunologic framework than a genetic one. The biomarker consistently tracked with features of allergic and eosinophilic inflammation, while the exploratory genetic analysis did not provide clinically actionable information. This distinction is important for interpretation: the present study supports the practical value of FeNO in phenotyping airway inflammation in children and adolescents with asthma, but does not support the routine clinical use of rs2228137 genotyping in this setting. Future studies with larger samples, longitudinal follow-up, and broader phenotypic characterization may help determine whether FCER2 variation has a context-specific role in selected patient subgroups, particularly regarding exacerbation risk, corticosteroid responsiveness, or more severe inflammatory phenotypes.

###  Limitations

Several limitations of this study should be acknowledged. First, the sample size was modest, particularly for genotype subgroup analyses. The rs2228137 TT genotype was present in only five patients, substantially limiting the statistical power to detect genotype–phenotype associations. Second, the cross-sectional design precludes causal inference and does not allow assessment of changes in FeNO, asthma control, or inflammatory markers over time; the conceptual framework in Figure 3 was used only to guide covariate selection. Third, all patients were on ICS-based therapy, which may have reduced inflammatory differences and limited the ability to identify genotype-related effects. Although the ICS daily-dose category and treatment duration are now reported, the exact ICS molecule, budesonide-equivalent dose, GINA step, treatment adherence and inhaler technique were not systematically recorded; because these factors strongly influence FeNO, their absence remains a limitation. Active/current tobacco exposure, allergic rhinitis and atopic dermatitis were likewise not systematically recorded and may independently affect FeNO. Fourth, the study population was recruited from a single tertiary center in Istanbul, which could affect how well the findings apply to community-based pediatric populations or other ethnic groups. Fifth, because no healthy control group was included, the study was not designed to assess genotype-related disease susceptibility. Sixth, although the primary inference rests on a single prespecified multivariable model, the adjustment set was limited to age, sex and BMI z-score, and residual confounding by unmeasured factors cannot be excluded. Finally, the descriptive analyses by FeNO and control category and the exploratory genotype analyses are hypothesis-generating; the latter were not corrected for multiple testing and their *p*-values should be interpreted cautiously.

## 5. Conclusions

In Turkish children and adolescents with asthma receiving ICS-based therapy, FeNO was independently associated with peripheral blood eosinophil percentage and symptom-based asthma control in a multivariable model adjusted for prespecified baseline covariates; total IgE and aeroallergen sensitization showed descriptive gradients across clinically defined FeNO categories but were not independently associated with FeNO. These findings support the role of FeNO as an adjunctive biomarker of type 2 airway inflammation in this population. The exploratory analysis of the FCER2 rs2228137 polymorphism did not reveal genotype–phenotype associations; given the small subgroups and limited statistical power, this exploratory finding should be regarded as hypothesis-generating. Larger multicenter longitudinal studies are needed to validate FeNO-based clinical and inflammatory profiling and to clarify the potential relevance of rs2228137 in pediatric asthma.

## Figures and Tables

**Figure 1:**
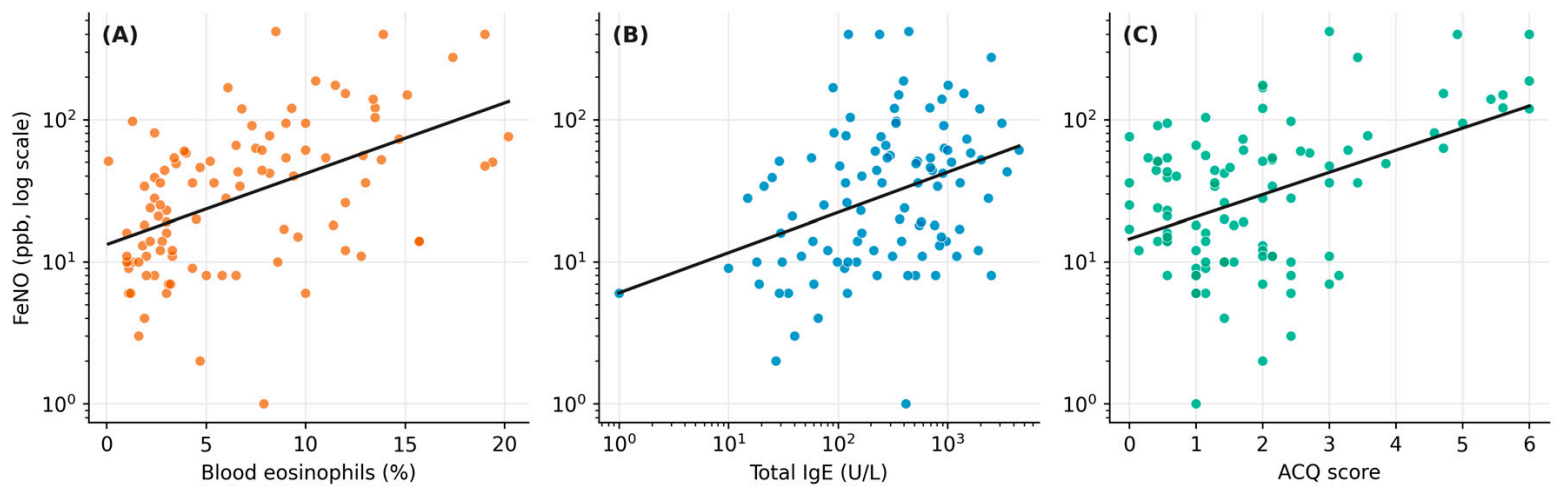
Relationships between FeNO (log scale) and (**A**) blood eosinophil percentage, (**B**) total IgE, and (**C**) ACQ score (*n* = 100), shown for descriptive purposes. Solid lines are least-squares trend fits on the log scale; the adjusted estimates from the primary multivariable model are given in the corresponding regression table below.

**Table 1:** General characteristics of study subjects (*n* = 100).

Baseline Characteristic	Value
Age, mean (±SD), years	12.2 ± 3.3 (range 6–18)
Male, *n* (%)	60 (60%)
BMI, mean (±SD), kg/m^2^	20.13 ± 4.04
History of atopy, *n* (%)	55 (55%)
Prenatal cigarette exposure, *n* (%)	48 (48%)
Family history of asthma, *n* (%)	40 (40%)
Peripheral blood eosinophil percentage, mean (±SD), %	6.49 ± 4.81
Total IgE, median (IQR), U/L	289 (67–851)
FeNO, median (IQR), ppb	35.0 (10.0–88.0)
FEV_1_, mean (±SD), % predicted	100.01 ± 15.83
FVC, mean (±SD), % predicted	98.21 ± 16.28
SPT positivity, *n* (%)	74 (74%)

Data are presented as mean ± SD, median (IQR) or *n* (%). BMI, body mass index; FeNO, fractional exhaled nitric oxide; FEV_1_, forced expiratory volume in 1 s; FVC, forced vital capacity; IgE, immunoglobulin E; IQR, interquartile range; SPT, skin prick test.

**Table 2:** Primary multivariable linear regression of ln(FeNO).

Predictor (Unit)	β	95% CI	*p*-Value
Blood eosinophils (per 1%)	0.082	0.039 to 0.124	<0.001
ln(total IgE) (per log-unit)	0.069	−0.090 to 0.229	0.39
SPT positive (vs. negative)	0.058	−0.448 to 0.565	0.82
ACQ score (per 1 unit)	0.264	0.135 to 0.394	<0.001
Age (per year)	0.074	0.013 to 0.134	0.02
Female (vs. male)	−0.090	−0.483 to 0.304	0.65
BMI z-score (per 1 SD)	−0.086	−0.248 to 0.076	0.29

Multivariable linear regression fitted to the natural logarithm of FeNO (ppb); *n* = 98 (participants with complete BMI z-score). R^2^ = 0.42, adjusted R^2^ = 0.38; F(7,90) = 9.4, *p* < 0.001. All VIF < 1.5. Blood eosinophils, ln(total IgE), SPT positivity and ACQ score are the clinical/inflammatory variables of interest; age, sex and BMI z-score are the prespecified baseline covariates. β denotes the change in ln(FeNO) per one-unit increase in the predictor, adjusted for all other variables in the model; the corresponding percentage change in FeNO is (e^β^ − 1) × 100. ACQ, Asthma Control Questionnaire; BMI, body mass index; CI, confidence interval; FeNO, fractional exhaled nitric oxide; IgE, immunoglobulin E; SPT, skin prick test; VIF, variance inflation factor.

**Table 3:** Clinical and laboratory parameters across ATS pediatric FeNO categories (descriptive).

Parameter	<20 ppb (*n* = 40)	20–35 ppb (*n* = 10)	>35 ppb (*n* = 50)
FEV_1_, mean (±SD), % predicted	101.9 ± 14.7	100.2 ± 7.5	98.7 ± 17.7
Peripheral blood eosinophil percentage, mean (±SD), %	5.0 ± 4.3	5.0 ± 3.2	7.8 ± 5.1
Total IgE, median (IQR), U/L	157.5 (28–450)	140.5 (42–780)	443 (89–1200)
ACQ score, median (IQR)	0.65 (0.28–1.28)	1.35 (0.75–1.80)	2.10 (1.35–3.14)
SPT positivity, *n* (%)	25 (62.5%)	7 (70.0%)	42 (84.0%)
rs2228137 TT genotype, *n* (%)	1 (2.5%)	2 (20.0%)	2 (4.0%)

Data are presented as mean ± SD, median (IQR) or *n* (%). Descriptive analysis for clinical interpretation of the ATS thresholds; no between-group significance tests were performed (see Section 2.8). Adjusted estimates are given in Table 2. ACQ: Asthma Control Questionnaire; ATS: American Thoracic Society; FeNO: fractional exhaled nitric oxide; FEV_1_: forced expiratory volume in 1 s; IgE: immunoglobulin E; SPT: skin prick test.

**Table 4:** Clinical and laboratory parameters across ACQ-defined asthma control groups (descriptive).

Parameter	Well-Controlled (*n* = 32)	Partially Controlled (*n* = 30)	Uncontrolled (*n* = 38)
Peripheral blood eosinophil percentage, mean (±SD), %	6.1 ± 4.9	5.9 ± 4.2	7.1 ± 5.3
Total IgE, median (IQR), U/L	273 (60–1050)	332.5 (55–980)	357.5 (98–1200)
FeNO, median (IQR), ppb	16 (9–42)	35 (11–73)	44 (21–90)
SPT positivity, *n* (%)	19 (59.4%)	21 (70.0%)	34 (89.5%)
rs2228137 TT genotype, *n* (%)	1 (3.1%)	1 (3.3%)	3 (7.9%)

Data are presented as mean ± SD, median (IQR) or *n* (%). Descriptive analysis; no between-group significance tests were performed (see Section 2.8). The ACQ score was analysed as a continuous variable in the primary multivariable model (Table 2). ACQ: Asthma Control Questionnaire; FeNO: fractional exhaled nitric oxide; IgE: immunoglobulin E; SPT: skin prick test.

**Table 5:** Exploratory analysis of clinical and laboratory parameters according to FCER2 rs2228137 genotype, with genotype entered as a covariable in adjusted regression models.

Parameter	CC Genotype (*n* = 60)	TC Genotype (*n* = 35)	TT Genotype (*n* = 5)	Codominant Model, *p* ^a^	Additive Model, *p* ^a^
FEV_1_, mean (±SD), % predicted	100.0 ± 14.0	101.0 ± 17.0	94.5 ± 21.1	0.61	0.94
Peripheral blood eosinophil percentage, mean (±SD), %	6.5 ± 5.1	6.6 ± 4.3	3.4 ± 2.0	0.62	0.54
Total IgE, median (IQR), U/L	289 (60–980)	330.5 (72–1100)	997 (200–2342)	0.71	0.52
FeNO, median (IQR), ppb	36 (12–76)	26 (11–50)	28 (28–58)	0.61	0.40
SPT positivity, *n* (%)	45 (75.0%)	25 (71.4%)	4 (80.0%)	0.59	0.54
Uncontrolled asthma (ACQ > 1.5), *n* (%)	23 (38.3%)	12 (34.3%)	3 (60.0%)	0.59	0.49

Data are presented as mean ± SD, median (IQR) or *n* (%). ^a^ *p*-values from regression models adjusted for age, sex and BMI z-score in which genotype was entered as a covariable (*n* = 98; *n* = 96 for FEV_1_): linear regression for FEV_1_, eosinophil percentage, ln(total IgE) and ln(FeNO); logistic regression for SPT positivity and uncontrolled asthma. Codominant coding: TC and TT versus CC (2-df Wald test; primary); additive coding: per T allele (0/1/2). Dominant and recessive coding gave similar results (all *p* ≥ 0.30). ACQ, Asthma Control Questionnaire; FeNO, fractional exhaled nitric oxide; FEV_1_, forced expiratory volume in 1 s; IgE, immunoglobulin E; IQR, interquartile range; SPT, skin prick test.

## Data Availability

The data that support the findings of this study are available from the corresponding author upon reasonable request. The data are not publicly available because they contain genetic and clinical information that could compromise the privacy of the paediatric participants, in accordance with the institutional ethics committee approval and applicable data-protection regulations.

## Supplementary Materials

The following supporting information can be downloaded at: https://www.mdpi.com/article/10.3390/children13101291/s1, Table S1: Sensitivity analysis: associations with FeNO category at pediatric versus adult ATS decision points (proportional-odds logistic regression).

## Abbreviations

ACQ	Asthma Control Questionnaire
ATS	American Thoracic Society
BMI	Body Mass Index
FCER2	Fc Fragment of IgE Receptor II (CD23)
FeNO	Fractional Exhaled Nitric Oxide
FEV1	Forced Expiratory Volume in 1 Second
FVC	Forced Vital Capacity
GINA	Global Initiative for Asthma
ICS	Inhaled Corticosteroid
IgE	Immunoglobulin E
IL	Interleukin
LABA	Long-Acting Beta-Agonist
PCR	Polymerase Chain Reaction
SNP	Single-Nucleotide Polymorphism
SPT	Skin Prick Test
CI	Confidence Interval
DAG	Directed Acyclic Graph
VIF	Variance Inflation Factor
