# Supplementary material for: Clinical and Inflammatory Correlates of Fractional Exhaled Nitric Oxide in Turkish Children and Adolescents with Asthma: Exploratory Analysis of the FCER2 rs2228137 Polymorphism
Source: Children (Basel). 2026 Sep 23;13(10):1291. doi: 10.3390/children13101291 (PMC13703703; doi:10.3390/children13101291)
Supplement: Supplementary file 1 [file children-13-01291-s001.zip › children-4446735-supplementary.pdf]

children

MDPI

Supplementary Materials

Table S1. Sensitivity analysis: associations with FeNO category at pediatric versus adult ATS decision points (proportional-odds logistic regression).

Covariable (unit)	Pediatric decision points (<20 / 20–35 / >35 ppb), unadjusted OR (95% CI); p	Pediatric decision points, adjusted OR (95% CI); p [a]	Adult decision points (<25 / 25–50 / >50 ppb), unadjusted OR (95% CI); p	Adult decision points, adjusted OR (95% CI); p [a]
Blood eosinophils (per 1%)	1.22 (1.11–1.35); <0.001	1.22 (1.10–1.34); <0.001	1.21 (1.11–1.32); <0.001	1.20 (1.10–1.31); <0.001
ln(total IgE) (per log-unit)	1.56 (1.18–2.06); 0.002	1.50 (1.12–2.01); 0.007	1.52 (1.16–1.99); 0.002	1.48 (1.11–1.97); 0.008
SPT positive (vs negative)	2.99 (1.22–7.35); 0.02	2.27 (0.89–5.79); 0.09	2.37 (0.98–5.73); 0.06	1.63 (0.64–4.16); 0.31
ACQ score (per 1 point)	1.69 (1.23–2.34); 0.001	1.72 (1.23–2.40); 0.002	1.90 (1.39–2.58); <0.001	1.97 (1.42–2.72); <0.001

Proportional-odds (ordinal logistic) regression of the three-level FeNO category on each covariable; ORs express the odds of being in a higher FeNO category per unit of the covariable. [a] *Adjusted for age, sex and BMI z-score (n = 98 with complete BMI z-score; unadjusted models n = 100). This sensitivity analysis is retained from the previous revision and is reported for completeness; the primary inference rests on the continuous-FeNO model in Table 2, which does not depend on the choice of decision points. ACQ, Asthma Control Questionnaire; ATS, American Thoracic Society; CI, confidence interval; FeNO, fractional exhaled nitric oxide; IgE, immunoglobulin E; OR, odds ratio; SPT, skin prick test.*
